# The use of dimethylsulfoxide as a solvent in enzyme inhibition studies: the case of aldose reductase

**DOI:** 10.1080/14756366.2017.1363744

**Published:** 2017-08-31

**Authors:** Livia Misuri, Mario Cappiello, Francesco Balestri, Roberta Moschini, Vito Barracco, Umberto Mura, Antonella Del-Corso

**Affiliations:** a Department of Biology, Biochemistry Unit, University of Pisa, Pisa, Italy;; b Tuscany Region PhD School in Biochemistry and Molecular Biology, Italy;; c Interdepartmental Research Center Nutrafood “Nutraceuticals and Food for Health”, University of Pisa, Pisa, Italy

**Keywords:** Dimethyl sulfoxide, aldose reductase, aldose reductase differential inhibitors

## Abstract

Aldose reductase (AR) is an enzyme devoted to cell detoxification and at the same time is strongly involved in the aetiology of secondary diabetic complications and the amplification of inflammatory phenomena. AR is subjected to intense inhibition studies and dimethyl sulfoxide (DMSO) is often present in the assay mixture to keep the inhibitors in solution. DMSO was revealed to act as a weak but well detectable AR differential inhibitor, acting as a competitive inhibitor of the L-idose reduction, as a mixed type of non-competitive inhibitor of HNE reduction and being inactive towards 3-glutathionyl-4-hydroxynonanal transformation. A kinetic model of DMSO action with respect to differently acting inhibitors was analysed. Three AR inhibitors, namely the flavonoids neohesperidin dihydrochalcone, rutin and phloretin, were used to evaluate the effects of DMSO on the inhibition studies on the reduction of L-idose and HNE.

## Introduction

Since its synthesis almost 150 years ago^1^, dimethyl sulfoxide (DMSO) has been proved to be a versatile molecule capable of accomplishing a variety of functions. The molecule has an incredible number of different uses due to its particular chemicophysical features^2^ ^,^ ^3^, chemical reactivity^4^, pharmacological effects^5–9^ and true or presumed therapeutic properties^10^ ^,^ ^11^.

Since DMSO is a polar aprotic solvent, it can dissolve both polar and hydrophobic compounds. In addition, as it can be mixed with water as well as with a number of organic solvents, it has been used as a vehicle for the delivery of various molecules in cultured cells and in *in vivo* experiments^12–15^. DMSO has often been used as a solvent for hydrophobic molecules to investigate their effects on aqueous media. Thus, many enzymes have been characterised for substrate specificity and susceptibility to inhibition using DMSO. Its ability to both activate and inhibit enzyme activity *in vitro* and *in situ* has also been reported^16–19^. When a molecular species, not necessarily connected to the enzymatic reaction, is present in the assay mixture, its effect should be ascertained and if necessary its concentration must be kept constant when other parameters (i.e. inhibitors and/or substrate concentrations) are varied. However, this good experimental practice, which should be adopted irrespectively of the known effects of the solvent, may be hindered as the concentration of DMSO in the assay is often undefined or indeterminable, or appears to change depending on the concentration of the inhibitor^20–26^.

Aldose reductase (AR), since its involvement in the onset of diabetic complications, has been the subject of intense study aimed at finding valuable inhibitors to control its activity^27^ ^,^ ^28^. Such studies often entail the use of DMSO in order to ensure the solubilisation of inhibitory molecules in the assay mixture. DMSO has also been used as a vehicle to enable AR inhibitors (ARIs) to enter target cells^12^. A recent new approach in the AR inhibition deals with the search of aldose reductase differential inhibitors (ARDIs), which should act depending on the substrate AR is working on, thus blocking the deleterious action of the enzyme and preserving its detoxifying action^29^ ^,^ ^30^.

This study on ARI shows evidence of a differential inhibitory action exerted by DMSO on the AR activity and examines its influence on the kinetic characterisation of AR inhibitors.

## Materials and methods

### Materials

Bovine serum albumin (BSA), D,L-dithiothreitol (DTT), D,L-glyceraldehyde (GAL), DMSO, EDTA, were purchased from Sigma-Aldrich (Saint Louis, MO). NADPH and L-idose were supplied by Carbosynth (Compton, England); YM10 ultrafiltration membranes were obtained from Merck-Millipore (Darmstadt, Germany); neohesperidin dihydrochalcone (NHDC), rutin and phloretin were obtained from Extrasynthese (Lyon, France). All other chemicals were of reagent grade.

### Assay of aldose reductase

The AR activity was determined at 37 °C as previously described^31^, following the decrease in absorbance at 340 nm due to NADPH oxidation (*ε*_340_ = 6.22 mM^−1^·cm^−1^) through a Biochrom Libra S60 spectrophotometer (Biochrom, Cambridge, United Kingdom). The standard assay mixture contained a 0.25 M sodium phosphate buffer pH 6.8, 0.18 mM NADPH, 0.4 M ammonium sulphate, 0.5 mM EDTA and 4.7 mM GAL. One unit of enzyme activity is the amount that catalyses the conversion of 1 µmol of substrate/min in the above assay conditions. These assay conditions were also adopted to assess the effectiveness of inhibitors when L-idose, trans-4-hydroxy-2,3-nonenal (HNE), or 3-glutathionyl-4-hydroxynonenal (GSHNE) was used as a substrate instead of GAL.

### Purification of human recombinant AR

The human recombinant AR (*h*AR) was expressed and purified as previously described^32^. The purity of the final enzyme preparation was assessed by SDS-PAGE^33^ and gels were stained with silver nitrate^34^, which showed a single band corresponding to a molecular weight of approximately 34 kDa. The specific activity of purified *h*AR was 5.3 U/mg. The purified enzyme was stored at −80 °C in a 10 mM sodium phosphate buffer pH 7.0 containing 2 mM DTT and 30% (w/v) glycerol. Before use, the enzyme was extensively dialysed against a 10 mM sodium phosphate buffer pH 7.0.

### Other methods

The protein concentration was determined according to Bradford^35^, using BSA as a standard protein. HNE was prepared as previously described^36^. GSHNE was prepared as previously described^37^ by incubation of GSH and HNE, monitoring the time course of GSH consumption. The residual GSH was determined as previously described^38^.

Statistical analysis was performed using the two-way ANOVA test operated with GraphPad version 6.0 software (GraphPad Software, La Jolla, CA).

## Results and discussion

### DMSO as differential inhibitor of hAR

**Figure 1.**
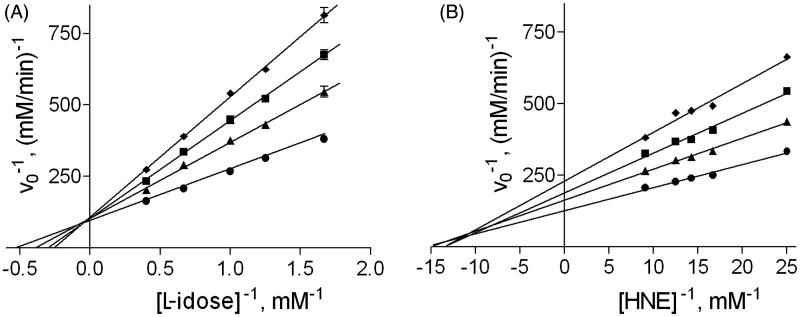
Effect of DMSO on aldose reductase activity. Inhibition data of DMSO on AR are reported in double reciprocal plots. *Panel A*: AR activity was determined using different L-idose concentrations, both in the absence (circle) and in the presence of 40 mM (triangle), 100 mM (square), and 200 mM (diamond) DMSO. *Panel B*: AR activity was determined using different HNE concentrations, both in the absence (circle) and in the presence of 100 mM (triangle), 200 mM (square), and 300 mM (diamond) DMSO.

The ability of DMSO to affect AR activity was evaluated using L-idose and HNE, two of the substrates routinely used to define the inhibitory features of ARDIs^29^ ^,^ ^30^ ^,^ ^39^. As shown in Figure 1, DMSO shows a competitive inhibition towards L-idose reduction and a mixed type of inhibition towards HNE reduction. In the case of L-idose as substrate, an inhibitory constant K_i_ (dissociation constant of the EI complex) of 235 ± 17 mM was determined, while for HNE a K_i_ of 266 ± 7 mM and a K_i_^′^ (dissociation constant of the ESI complex) of 378 ± 24 mM were measured. Although the overall inhibitory power on the two different reactions appears to be essentially the same, DMSO basically behaves as a differential inhibitor, since it discriminates between the two different substrates undergoing reduction. The effect of DMSO was also tested on GSHNE, an HNE derivative recognised as a substrate by AR, whose reduction product is known to elicit the inflammation response through the activation of the NF-kB cascade^40^. In this case, no effect of interference on the enzyme activity was observed up to 100 mM of DMSO in the assay mixture in the presence of different substrate concentrations. Similarly, no effect of DMSO was evident on the reduction of GAL catalysed by AR.

### *In vitro* effect of DMSO in the AR inhibition study

In order to evaluate the possible influence of DMSO in identifying ARDIs, the possibility that an ARI acts differently on the reduction of different substrates was also considered. Thus, three different ARIs, namely the flavonoids neohesperidin dihydrochalcone (NHDC), rutin and phloretin, were used to evaluate the effect of DMSO in the assay mixture when the inhibition features of these molecules were evaluated in the reduction of either L-idose or HNE. This experimental approach was possible due to the solubility of the above inhibitors in 0.7% (v/v) methanol (approximately 0.17 M). At this concentration, the methanol in the enzyme assay mixture did not affect the AR activity (an inhibition less than 5% was observed) in the range of substrate concentrations of 0.4–4 mM and 40–110 µM for L-idose and HNE, respectively.

**Figure 2.**
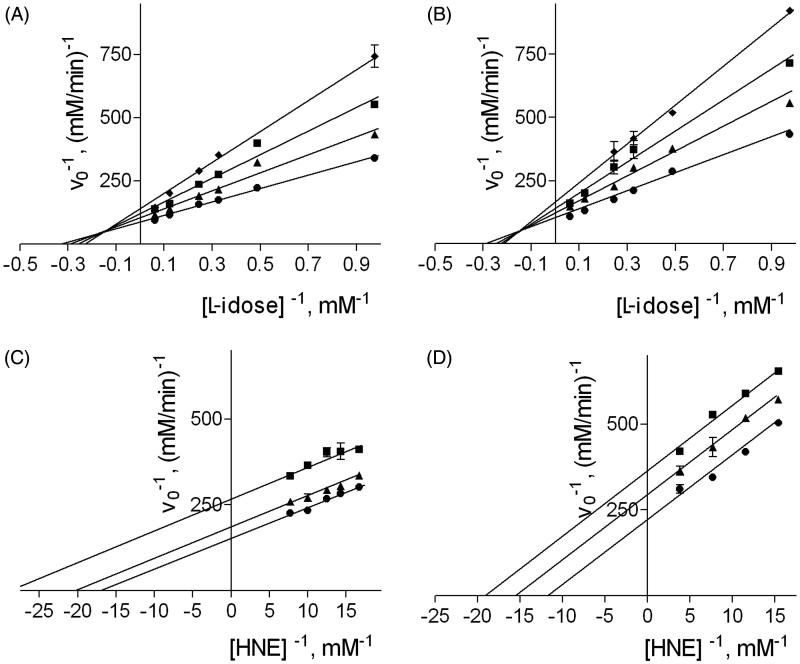
Effect of DMSO on the inhibition of AR by NHDC. *Panel A* and *B*: AR activity was determined using different L-idose concentrations in the presence of 0 (circle), 50 (triangle), 100 (square), or 150 (diamond) µM NHDC, both in the absence (*Panel A*) and in the presence of 100 mM DMSO (*Panel B*). AR activity was determined using different HNE concentrations, both in the presence of 0 (circle), 50 (triangle), and 100 (square) µM NHDC both in the absence (*Panel C*) and in the presence of 200 mM DMSO (*Panel D*).

Figure 2 reports the results of a typical kinetic study aimed at determining the dissociation constants K_i_ and K_i_^′^ of the binary (enzyme:inhibitor) and the ternary (enzyme:substrate:inhibitor) complexes, respectively, for NHDC, used as an inhibitor of the reduction of both L-idose and HNE. The same analysis was performed with phloretin and rutin (data not shown). Table 1 reports the K_i_ and K_i_^′^ values of the three inhibitors measured for the reduction of both L-idose and HNE. While phloretin showed essentially the same inhibitory activity towards both substrates, rutin and NHDC exerted a modest, differential inhibitory action on L-idose reduction with respect to HNE reduction. In fact, both rutin and NHDC behave as mixed inhibitors of AR in the presence of L-idose, and as uncompetitive inhibitors in the presence of HNE. While for rutin, the ability to interact with the AR:L-idose complex prevailed, NHDC appeared to preferentially bind the free enzyme.

**Table 1.** Inhibition constants of neohesperidin dihydrochalcone, rutin and phloretin for the reduction of L-idose, or HNE determined at different DMSO concentrations.

			K_i_ (µM)				K_i_′ (µM)			
Substrate	Inhibitor	Inhibition model	No DMSO	DMSO 40 mM	DMSO 100 mM	DMSO 200 mM	No DMSO	DMSO 40 mM	DMSO 100 mM	DMSO 200 mM
L-idose	NHDC	Mixed	93 ± 17	114 ± 15	125 ± 16	194 ± 17	292 ± 17	286 ± 53	230 ± 35	283 ± 2
HNE	NHDC	Uncompetitive	–	–	–	–	122 ± 19	134 ± 11	150 ± 22	199 ± 6
L-idose	Rutin	Mixed	17.8 ± 3.0	20.3 ± 1.1	24.8 ± 3.8	45.6 ± 5.5	9.3 ± 1.1	7.6 ± 1.2	11.7 ± 0.5	12.7 ± 2.0
HNE	Rutin	Uncompetitive	–	–	–	–	9.2 ± 0.9	10.5 ± 2.0	12.8 ± 1.9	16.5 ± 1.9
			–	–	–	–				
L-idose	Phloretin	Mixed	66.6 + 5.8	85.4 + 9.0	98.9 + 11.7	120.8 + 43.1	52.5 + 3.8	62.9 + 2.3	50.3 + 4.8	60.9 + 2.1
HNE	Phloretin	Mixed	60.5 + 7.2	79.0 + 23.5	94.8 + 12.1	106.8 + 12.3	56.5 + 4.5	64.7 + 1.4	73.4 + 7.5	99.5 + 7.3

To evaluate the effect of DMSO on the inhibitory action of the three flavonoids, the same analytical approach was performed for the three inhibitors acting on both L-idose and HNE reduction, with DMSO in different concentrations in the assay mixture. The values of the apparent K_i_ and K_i_^′^ are again reported in Table 1. In the case of L-idose reduction, DMSO led to an increase in K_i_ values, while K_i_^′^ values remained essentially unchanged for all the three inhibitors. However in terms of HNE reduction, DMSO caused an increase in K_i_^′^ values (approximately twice at the maximal DMSO concentration) for rutin and NDHC, and a similar increase in both kinetic inhibition constants for phloretin.

### Kinetic models of double inhibition enzymes

In order to explain the effects of the observed interference of DMSO on the kinetic characterisation of differently acting ARIs, a kinetic analysis of different inhibition models was considered. Thus the effects were analysed of a competitive inhibition or a mixed type of inhibition (as is the case of DMSO towards the reduction of L-idose or HNE, respectively) on a competitive, uncompetitive, and mixed type of inhibition of the molecules under investigation.

**Figure 3.**
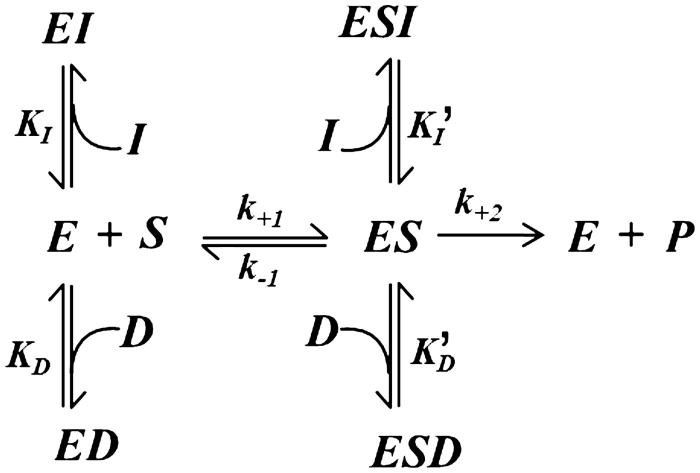
Kinetic model of mutual exclusion inhibition by DMSO and a generic inhibitor on the transformation of a generic substrate. See the text for an explanation of the symbols.

The analysis was performed according to a previously reported double inhibition approach^41^ considering a simple mutual exclusion kinetic model (Figure 3) in which both DMSO (D) and a generic inhibitor of the enzyme (I) behave as mixed type inhibitors with respect to the AR catalysed reduction of a generic substrate S.

Thus, the general equation for the initial reaction rate as a function of the substrate concentration:

$$v_{0}=k_{+2}\left[ES\right]$$

taking into account the steady state conditions for the substrate transformation:

$$k_{+1}\left[E\right]\left[S\right]=\left(k_{-1}+k_{+2}\right)\left[ES\right]$$

the equilibrium conditions for the enzyme targeting by the inhibitors:

$$K_{D}=\frac{\left[E\right]\left[D\right]}{\left[ED\right]};\;K_{I}=\frac{\left[E\right]\left[I\right]}{\left[EI\right]};\;K_{D}^{\prime }=\frac{\left[ES\right]\left[D\right]}{\left[ESD\right]};\;K_{I}^{\prime }=\frac{\left[ES\right]\left[I\right]}{\left[ESI\right]}$$

and the mass balance for the enzyme

$$\left[E_{T}\right]=\left[E\right]+\left[ES\right]+\left[ED\right]+\left[EI\right]+\left[ESD\right]+\left[ESI\right]$$

can be developed into:

$$\frac{v_{0}}{\left[E_{T}\right]}=\frac{k_{+2}}{1+\frac{K_{M}}{\left[S\right]}+\frac{K_{M}\left[D\right]}{K_{D}\left[S\right]}+\frac{K_{M}\left[I\right]}{K_{I}\left[S\right]}+\frac{\left[D\right]}{K_{D}^{\prime }}+\frac{\left[I\right]}{K_{I}^{\prime }}}$$

in which *K_M_* stands for the Michaelis constant. This equation, after simple algebra, can be represented in the usual form of a rectangular hyperbola.

(1) $$v_{0}=\frac{\frac{k_{+2}\left[E_{T}\right]\left[S\right]}{1+\frac{\left[D\right]}{K_{D}^{\prime }}+\frac{\left[I\right]}{K_{I}^{\prime }}}}{K_{M}\left(\frac{1+\frac{\left[D\right]}{K_{D}}+\frac{\left[I\right]}{K_{I}}}{1+\frac{\left[D\right]}{K_{D}^{\prime }}+\frac{\left[I\right]}{K_{I}^{\prime }}}\right)+\left[S\right]}$$

**Figure 4.**
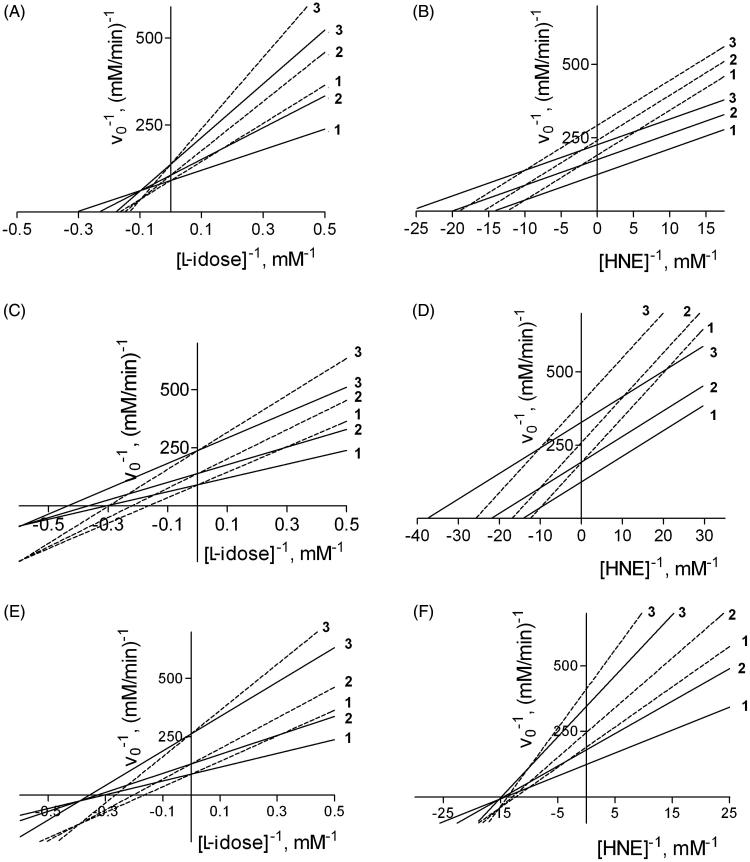
Simulation of the effect of DMSO on the inhibition of AR by NHDC, rutin, and phloretin. A computer-assisted plot was generated to simulate the effect of DMSO, described by Equation (1), on the inhibition of AR, using both L-idose (*left Panels*) and HNE (*right Panels*) as substrate. The kinetic parameters adopted in the simulation were as follows: i) V_MAX_ and K_M_ for L-idose as substrate were 0.011 mM/min and 2.3 mM, respectively; ii) V_MAX_ and K_M_ for HNE as substrate were 0.008 mM/min and 0.07 mM, respectively; iii) the inhibition kinetic parameters (i.e. K_i_ and K_i_^'^) referring to DMSO and to the three tested inhibitors are reported in Table 1. In each panel, curves 1, 2, and 3 were obtained both in the absence (solid line) and in the presence of 200 mM (dashed line) DMSO at the following inhibitor concentrations: NHDC 0, 50, and 100 µM, respectively (*Panels A* and *B*), rutin 0, 5, and 15 µM, respectively (*Panels C* and *D*), phloretin 0, 25, and 100 µM, respectively (*Panels E* and *F*).

This kinetic equation can be then simplified for different combinations of inhibition models referring both to DMSO and to the tested inhibitors (see Appendix). On the basis of Equation (1), the effect of DMSO on the kinetic behaviour of some inhibitors was evaluated by computer simulation (Figure 4). The kinetic parameters used in the simulation are those from the above inhibition kinetic study on DMSO towards both L-idose and HNE reduction (Figure 1) together with those referring to the three ARIs characterised above, which act in the absence of DMSO on either L-idose or HNE reduction (Table 1). Thus, Panels A, C, and E on the left in Figure 4 refer to the effect of DMSO (acting as a competitive inhibitor) on the reduction of L-idose in the presence of mixed type inhibitors, i.e. NDHC, rutin and phloretin, respectively (see Appendix, section 3). Similarly, Panels B, D and F on the right in Figure 4 refer to the effect of DMSO (acting as a mixed inhibitor) on the reduction of HNE in the presence of uncompetitive inhibitors (i.e. NDHC and rutin, Panels B and D, respectively), as depicted in the Appendix (section 4), and in the presence of a mixed type inhibitor (i.e. phloretin, Panel F), as reported in the general inhibition model (Figure 3 and Equation (1)).

Through this approach, using different DMSO concentrations (namely 40, 100, and 200 mM), it was possible to estimate the theoretical overall effect of DMSO on the apparent efficiency and on the inhibitors model of action (Table 2), and compare these results with the parameters in Table 1. This shows how the measured parameters are satisfactorily verified by the theoretical predictions emerging from the general kinetic model in Figure 3. In addition, the apparent linear fitting of the experimental data on double reciprocal plots suggest the occurrence of a double inhibition by the DMSO and the tested inhibitor, devoid of the simultaneous presence of both molecular species in the enzyme.

**Table 2.** Inhibition constants of neohesperidin dihydrochalcone, rutin and phloretin for the reduction of L-idose or HNE obtained through a computer assisted simulation at different DMSO concentrations.

			K_i_ (µM)			K_i_′ (µM)		
Substrate	Inhibitor	Inhibition model	DMSO 40 mM	DMSO 100 mM	DMSO 200 mM	DMSO 40 mM	DMSO 100 mM	DMSO 200 mM
L-idose	NHDC	Mixed	109	133	172	292	292	292
HNE	NHDC	Uncompetitive	–	–	–	135	155	187
								
L-idose	Rutin	Mixed	20.8	25.4	33.0	9.3	9.3	9.3
HNE	Rutin	Uncompetitive	–	–	–	10.2	11.6	14.1
			–	–	–			
L-idose	Phloretin	Mixed	77.9	95.0	123.3	52.5	52.5	52.5
HNE	Phloretin	Mixed	69.6	83.3	105.9	62.5	71.5	86.4

## Conclusions

These results highlight the need to keep the solvent rigorously constant in the enzyme assay mixture, and more importantly, suggest that the contribution of the DMSO used as solvent in AR inhibition studies may be not negligible in determining both the binding efficiency and the targeting model of the inhibitor. Regarding the importance of the DMSO effect as a solvent in inhibition studies, it is worth considering that when the effectiveness of the inhibitor under investigation is high, the low concentration needed to define the inhibition parameters may minimise the interference of the solvent. This is both due to mechanistic reasons, linked to the comparison of the kinetic parameters of the solvent and the inhibitor, and because the reduced level of the solvent needed to maintain the inhibitor in solution at low concentrations. However, when a significant level of the solvent is required to keep the molecule in solution, especially when the strength of the inhibitor is not extraordinarily high, the effect of the solvent cannot be disregarded. This is possibly the case in the search and characterisation of AR differential inhibitors^30^. In fact, since these molecules are required in order to inhibit the enzyme depending on the substrate is undergoing to enzymatic transformation, their potency may not be very high. All this simply emerges viewing DMSO as an external additional independent factor with respect to the mechanism of the enzymatic reaction and to its inhibition.

More concerns may arise when considering the possible participation of the solvent in the targeting event of the enzyme by the inhibitor. In fact, the features of the inhibitor may be strongly affected by the kinetics and thermodynamic restrictions of the desolvation step of the inhibitor from DMSO to the enzyme interactive site. This kind of unavoidable problem, which generally occurs in poorly water-soluble ligand studies, has no easy solution. Only a comparative study of the specific inhibitor in different solvents or solvent cocktails might provide an insight into the role of DMSO in the effectiveness and inhibition mode of an inhibitory molecule.

## Appendix

On the basis of the same relationships and restrictions adopted in Figure 3 (see text), and taking into account the appropriate enzyme mass balance, the general kinetic equation:

$$v_{0}=k_{+2}\left[ES\right]$$

can be reformulated, considering the combination of different inhibition models of action of DMSO (D) and a generic inhibitor (I) with respect to the transformation of the substrate S:

**1) *Double inhibition model with both DMSO and I acting as competitive inhibitors***

From the enzyme mass balance:

$$\left[E_{T}\right]=\left[E\right]+\left[ES\right]+\left[ED\right]+\left[EI\right]$$

it follows

$$v_{0}=\frac{k_{+2}\left[E_{T}\right]\left[S\right]}{K_{M}\left(1+\frac{\left[D\right]}{K_{D}}+\frac{\left[I\right]}{K_{I}}\right)+\left[S\right]}$$

**2) *Double inhibition model with DMSO acting as competitive inhibitor and I acting as uncompetitive inhibitor***

From the enzyme mass balance:

$$\left[E_{T}\right]=\left[E\right]+\left[ES\right]+\left[ED\right]+\left[ESI\right]$$

it follows

$$v_{0}=\frac{\frac{k_{+2}\left[E_{T}\right]\left[S\right]}{1+\frac{\left[I\right]}{K_{I}^{\prime }}}}{K_{M}\left(\frac{1+\frac{\left[D\right]}{K_{D}}}{1+\frac{\left[I\right]}{K_{I}^{\prime }}}\right)+\left[S\right]}$$

**3) *Double inhibition model with DMSO acting as competitive inhibitor and I acting as mixed inhibitor***

From the enzyme mass balance:

$$\left[E_{T}\right]=\left[E\right]+\left[ES\right]+\left[ED\right]+\left[EI\right]++\left[ESI\right]$$

it follows:

$$v_{0}=\frac{\frac{k_{+2}\left[E_{T}\right]\left[S\right]}{1+\frac{\left[I\right]}{K_{I}^{\prime }}}}{K_{M}\left(\frac{1+\frac{\left[D\right]}{K_{D}}+\frac{\left[I\right]}{K_{I}}}{1+\frac{\left[I\right]}{K_{I}^{\prime }}}\right)+\left[S\right]}$$

**4) *Double inhibition model with DMSO acting as mixed inhibitor and I acting as uncompetitive inhibitor***

From the enzyme mass balance:

$$\left[E_{T}\right]=\left[E\right]+\left[ES\right]+\left[ED\right]+\left[ESD\right]+\left[ESI\right]$$

it follows

$$v_{0}=\frac{\frac{k_{+2}\left[E_{T}\right]\left[S\right]}{1+\frac{\left[D\right]}{K_{D}^{\prime }}+\frac{\left[I\right]}{K_{I}^{\prime }}}}{K_{M}\left(\frac{1+\frac{\left[D\right]}{K_{D}}}{1+\frac{\left[D\right]}{K_{D}^{\prime }}+\frac{\left[I\right]}{K_{I}^{\prime }}}\right)+\left[S\right]}$$
